# Lycopene Inhibits Oxidative Stress-Mediated Inflammatory Responses in Ethanol/Palmitoleic Acid-Stimulated Pancreatic Acinar AR42J Cells

**DOI:** 10.3390/ijms22042101

**Published:** 2021-02-20

**Authors:** Jaeeun Lee, Joo Weon Lim, Hyeyoung Kim

**Affiliations:** Department of Food and Nutrition, BK21 FOUR, College of Human Ecology, Yonsei University, Seoul 03722, Korea; jaeeun.chris@gmail.com (J.L.); jwlin11@yonsei.ac.kr (J.W.L.)

**Keywords:** lycopene, ethanol, palmitoleic acid, pancreatitis, reactive oxygen species

## Abstract

High alcohol intake results in the accumulation of non-oxidative ethanol metabolites such as fatty acid ethyl esters (FAEEs) in the pancreas. High FAEE concentrations mediate pancreatic acinar cell injury and are associated with alcoholic pancreatitis. Treatment with ethanol and the fatty acid palmitoleic acid (EtOH/POA) increased the levels of palmitoleic acid ethyl ester and induced zymogen activation and cytokine expression in pancreatic acinar cells. EtOH/POA induces nicotinamide adenine dinucleotide phosphate (NADPH) oxidase-mediated reactive oxygen species (ROS) production and pancreatic acinar cell injury. Lycopene, a bright-red carotenoid, is a potent antioxidant due to its high number of conjugated double bands. This study aimed to investigate whether lycopene inhibits the EtOH/POA-induced increase in ROS production, zymogen activation, and expression of the inflammatory cytokine IL-6 in EtOH/POA-stimulated pancreatic acinar AR42J cells. EtOH/POA increased the ROS levels, NADPH oxidase and NF-κB activities, zymogen activation, IL-6 expression, and mitochondrial dysfunction, which were inhibited by lycopene. The antioxidant N-acetylcysteine and NADPH oxidase 1 inhibitor ML171 suppressed the EtOH/POA-induced increases in ROS production, NF-κB activation, zymogen activation, and IL-6 expression. Therefore, lycopene inhibits EtOH/POA-induced mitochondrial dysfunction, zymogen activation, and IL-6 expression by suppressing NADPH oxidase-mediated ROS production in pancreatic acinar cells.

## 1. Introduction

Acute pancreatitis is an unpredictable and potentially lethal disease, characterized by a local and systemic inflammatory response [1]. Most patients present with mild acute pancreatitis, which is self-limiting and usually resolves within 1 week. Approximately 20% of patients develop moderate or severe acute pancreatitis, with necrosis of the pancreatic or peripancreatic tissue or organ failure, or both, and a substantial mortality rate of 20–40% [2]. Despite improvements in treatment and critical care, severe acute pancreatitis is still associated with high mortality rates. The three most common causes of acute pancreatitis are gallstone/biliary related, alcohol related, and idiopathic. Biliary pathology was estimated to be 28–45% of the cases while alcohol accounted for 19–41% of the cases [2,3].

In acute pancreatitis, premature or inappropriate activation of the protease precursor trypsinogen to trypsin can result in autodigestion of the pancreas [4]. Although trypsin-mediated cell death leads to pancreatic injury in early stages of pancreatitis, activation of inflammatory cascades, endoplasmic reticulum stress, autophagy, and mitochondrial dysfunction in the acinar cells are important in driving the profound systemic inflammatory response and extensive pancreatic injury [5].

In the pathogenesis of alcohol-related acute pancreatitis, the pancreas uses both oxidative and non-oxidative ethanol metabolism to degrade alcohol. Acetaldehyde, an oxidative metabolite of ethanol, is primarily generated in the liver and appears at an extremely low level in the circulation. However, non-oxidative metabolites such as fatty acid ethyl esters (FAEEs), which combine ethanol with fatty acids, accumulate in the pancreas [6,7]. Earlier, Laposata and Lange [8] showed that acetaldehyde cannot account for damage in organs such as the pancreas, heart, or brain, where oxidative metabolism is minimal or absent. They described FAEEs as toxic nonoxidative ethanol products which may be important in the pathophysiology of ethanol-induced disease in humans. Criddle et al. [9] demonstrated that FAAEs cases pancreatic Ca^2+^ overload via the inositol triphosphate receptor, depleted ATP, and depolarized mitochondria in the pancreatic acinar cells. Impaired mitochondrial ATP production induces calcium-ATPase pump failure, which results in sustained rise in Ca^2+^ in pancreatic acinar cells. These studies suggest a vicious cycle of Ca^2+^ mitochondrial dysfunction—loss of ATP synthesis in pancreatic acinar cells exposed to FAEEs. Voronina et al. [10] demonstrated that when cells rely on nonoxidative ATP production, FAEEs such as palmitoleic acid ethyl ester, diminish cytosolic and mitochondrial ATP levels in pancreatic acinar cells. Experimentally, treatment with ethanol in combination with the fatty acid palmitoleic acid (EtOH/POA) increased the levels of palmitoleic acid ethyl ester and induced acute pancreatic damage [11]. Therefore, EtOH/POA treatment has been used as a cellular model of alcohol-induced pancreatitis.

Reactive oxygen species (ROS) are associated with the pathogenesis of pancreatitis. Nicotinamide adenine dinucleotide phosphate (NADPH) oxidase, a membrane-bound enzyme complex, catalyzes the generation of ROS and serves as the most powerful source of endogenous ROS production [12]. Overproduction of ROS affects mitochondrial dysfunction by disrupting mitochondrial membrane potential (MMP), leading to ATP depletion in pancreatic acinar cells [13]. ROS can also serve as an intracellular second messenger involving the activation of nuclear factor kappa B (NF-κB) and the expression of cytokines. NF-κB, an inducible transcription factor, is associated with the regulation of inflammatory genes and subsequent inflammatory responses in acute pancreatitis [14]. We recently showed that EtOH/POA induces NADPH oxidase activation, ROS production, mitochondrial dysfunction, and pancreatic acinar cell injury [15].

High alcohol intake subjects pancreatic acinar cells to high levels of stress, resulting in premature activation of digestive enzymes. Inappropriate activation of trypsin within the pancreas is affected by many parameters, including Ca^2+^ levels and oxidative stress. Pancreatic acinar cells have digestive enzymes packed as inactive proenzymes in zymogen granules, and premature trypsin activation can be induced by high concentrations of FAEEs in the pancreas [16]. Prematurely activated digestive enzymes lead to acinar cell injury, since trypsin cleaves and activates other zymogens, including trypsinogen [17]. NADPH oxidase and ROS are known to be involved in zymogen activation and acute pancreatitis [18]. Raraty et al. [19] showed that a physiological cholecystokinin (CCK) level (10 pM) eliciting regular Ca^2+^ spiking did not evoke intracellular trypsin activation or vacuole formation. However, stimulation with 10 nM CCK, evoking a sustained rise in Ca^2+^, induced pronounced trypsin activation and extensive vacuole formation, both localized in the apical pole of pancreatic acinar cells. Taken together, NADPH oxidase activation to induce large amounts of ROS production and elevated Ca^2+^ mediate zymogen activation and vacuole formation which results in pancreatic damage.

Lycopene, which contributes to the red color of tomatoes, is a potent antioxidant and singlet oxygen quencher among carotenoids. Antioxidant activity of lycopene is linked to anti-inflammatory efficiency in various experimental pancreatitis models. Previously, we showed that lycopene reduces ROS levels, suppresses activation of transcription factor NF-κB, and thus inhibits inflammatory cytokine expression in cerulein-stimulated AR42J cells [20,21]. Lycopene showed protective effects against severe acute pancreatitis in rats through reducing ROS levels in pancreas and serum levels of damage-related molecular patterns (tumor necrosis factor-α, IL-6, macrophage inflammatory protein-1α, and monocyte chemotactic protein-1) [22]. In the rats with arginine-induced acute pancreatitis, lycopene treatment reduced tumor necrosis factor-α and myeloperoxidase activity, and down-regulated inducible nitric oxide synthase gene expression in pancreatic tissues. Pancreatic nitric oxide concentration was reduced and pancreatic glutathione (GSH) was increased in the lycopene group. Serum α-amylase and lipase activities were reduced by lycopene treatment [23]. Since oxidative stress plays a critical role in the pathogenesis of alcoholic pancreatitis, lycopene, which ameliorates oxidative stress, may be beneficial for reducing the symptoms or development of alcoholic pancreatitis.

The aims of the present study were as follows: (1) to determine whether EtOH/POA induces zymogen activation and IL-6 expression, which is mediated by NADPH oxidase-mediated ROS production in pancreatic acinar AR42J cells; and (2) to determine whether lycopene inhibits mitochondrial dysfunction, zymogen activation, and IL-6 expression by suppressing NADPH oxidase-mediated production of ROS in AR42J cells. In addition, to determine the involvement of ROS and NADPH oxidase in EtOH/POA-induced alterations (NF-κB activation, zymogen activation, and IL-6 expression), the cells were treated with the antioxidant N-acetylcysteine (NAC) and the NADPH oxidase 1 inhibitor ML171 prior to treatment with EtOH/POA.

## 2. Results

### 2.1. EtOH/POA Increases Trypsin Activity, NF-κB DNA-Binding Activity, and IL-6 Expression in AR42J Cells

**Figure 1:**
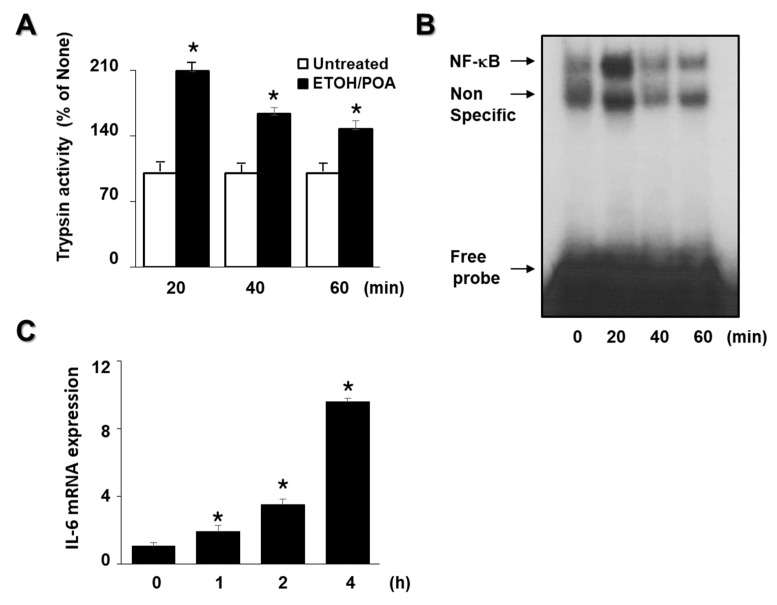
Ethanol and palmitoleic acid (EtOH/POA) increases trypsin activity, NF-κB DNA-binding activity, and IL-6 mRNA expression in AR42J cells. The cells were stimulated with EtOH/POA for 60 min (**A**,**B**) or 4 h (**C**). (**A**) Trypsin activity was assayed using a fluorogenic assay with a trypsin-specific substrate. (**B**) NF-κB DNA-binding activity was determined using an electrophoretic mobility shift assay (EMSA). (**C**) mRNA expression of IL-6 was determined by real-time polymerase chain reaction (PCR) and normalized to β-actin. The IL-6 mRNA level at 0 h was set as 1. Data were expressed as the mean ± standard error (SE) of three different experiments. For each experiment, the number of samples in each group was 4 (*n* = 4 per each group). * *p* < 0.05 vs. untreated cells (**A**) or 0 h (**C**).

To determine whether EtOH/POA increases trypsin activity, NF-κB DNA-binding activity, and IL-6 expression in AR42J cells, the cells were treated with EtOH/POA for 60 min (for the determination of trypsin activity and NF-κB DNA-binding activity) and 4 h (for the determination of IL-6 mRNA level). As shown in Figure 1A,B, EtOH/POA treatment increased both tyrpsin activity and NF-κB DNA-binding activity at 20 min which was reduced until 60 min. EtOH/POA induced IL-6 mRNA expression time-dependently until 4 h (Figure 1C). Therefore, a 20-min culture was used for the effect of lycopene on EtOH/POA-induced increases in tyrpsin activity and NF-κB DNA-binding activity. For the effect of lycopene on IL-6 mRNA expression in EtOH/POA-stimulated AR42J cells, a 4-h culture period was used.

### 2.2. Lycopene Inhibits Increases in ROS Levels, NADPH Oxidase Activity, and NF-κB DNA-Binding Activity in EtOH/POA-Stimulated AR42J Cells

**Figure 2:**
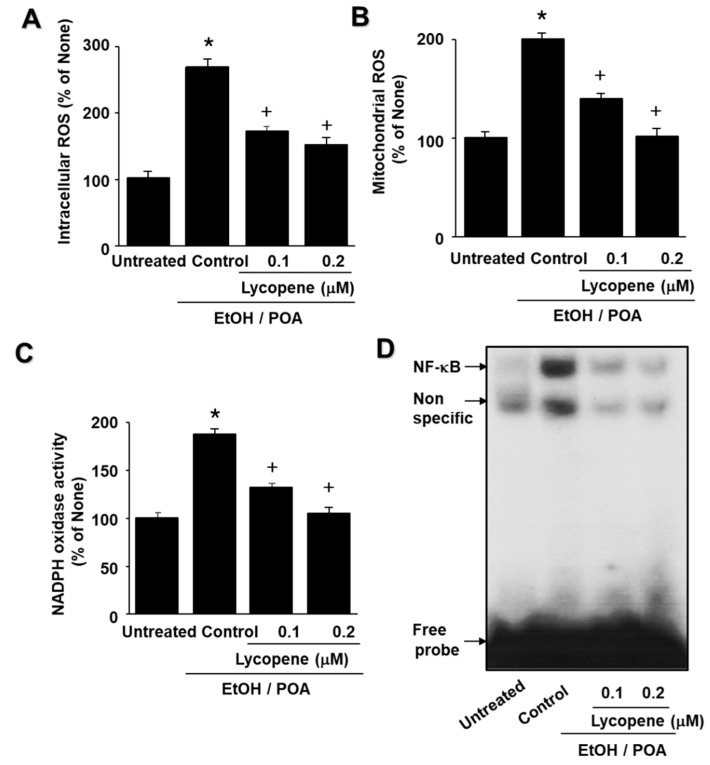
Lycopene inhibits increases in intracellular and mitochondrial reactive oxygen species (ROS), NADPH oxidase activity, and NF-κB DNA-binding activity in ethanol and palmitoleic acid (EtOH/POA)-stimulated AR42J cells. The cells were pretreated with the indicated concentrations of lycopene for 2 h and then stimulated with EtOH/POA for 10 min (**A**–**C**) or 20 min (**D**). (**A**) Intracellular ROS levels were determined by dichlorofluorescein (DCF) fluorescence. (B) Mitochondrial ROS levels were measured by determining the level of fluorescent MitoSOX. (C) NADPH oxidase activity was determined by lucigenin assay. (**A**–**C**) ROS level or NADPH oxidase activity in untreated cells was set as 100%. Data were expressed as the mean ± standard error (SE) of three different experiments. For each experiment, the number of samples in each group was 4 (*n* = 4 per each group). * *p* < 0.05 vs. untreated cells; + *p* < 0.05 vs. cells with EtOH/POA stimulation alone (control). (**D**) NF-κB DNA-binding activity was determined using an electrophoretic mobility shift assay (EMSA).

EtOH/POA increased the intracellular and mitochondrial ROS levels in AR42J cells, which was dose-dependently inhibited by lycopene (Figure 2A,B). NADPH oxidase is known to be the main source of ROS production in pancreatic acinar cells. To investigate whether lycopene inhibits EtOH/POA-induced NADPH oxidase activation, NADPH oxidase activity was determined using a lucigenin assay. Lycopene suppressed NADPH oxidase activity in EtOH/POA-stimulated cells in a dose-dependent manner (Figure 2C). Since ROS induce NF-κB activation, we determined whether EtOH/POA increases NF-κB DNA-binding activity and whether lycopene inhibits EtOH/POA-induced NF-κB activation in AR42J cells. As shown in Figure 2D, EtOH/POA increased the DNA-binding activity of NF-κB, which was reduced by lycopene treatment.

### 2.3. Lycopene Inhibits EtOH/POA-Induced Mitochondrial Dysfunction in AR42J Cells

**Figure 3:**
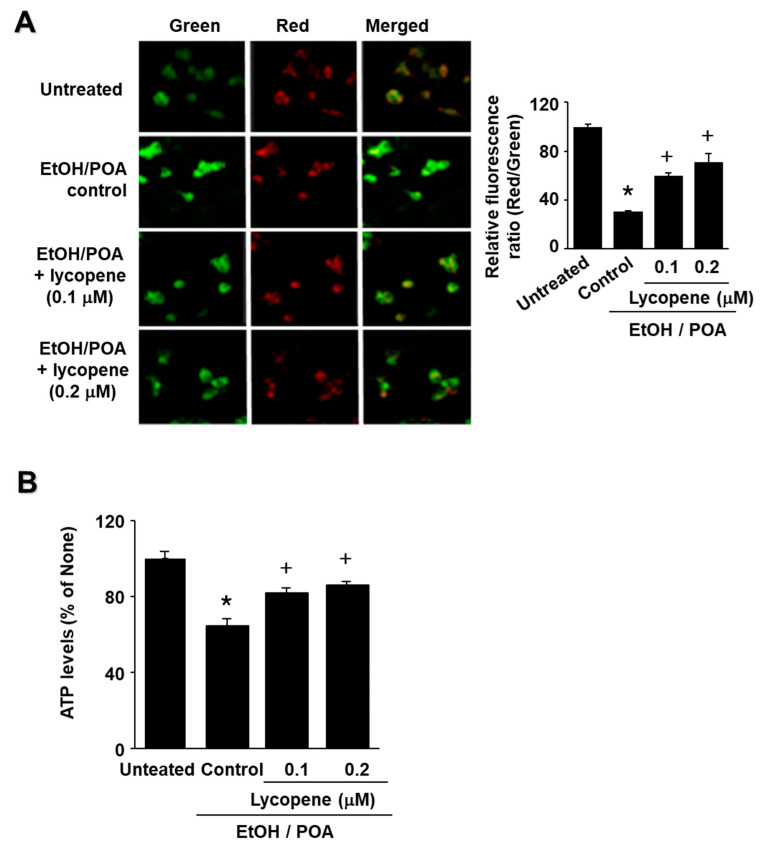
Lycopene inhibits ethanol and palmitoleic acid (EtOH/POA)-induced decreases in the mitochondrial membrane potential (MMP) and ATP levels in AR42J cells. (**A**) The cells were pretreated with the indicated concentrations of lycopene for 2 h and stimulated with EtOH/POA for 10 min. The cells were stained with JC-1 dye and visualized with a confocal laser scanning microscope (left panel). The MMP was determined by measuring the intensity of red emission relative to the intensity of green emission (right panel). A decrease in the red/green fluorescence intensity ratio indicates mitochondrial depolarization. (**B**) ATP levels were quantified using luminescent ATP substrate. The ATP concentration was determined by interpolating within the ATP standard reference. Data are expressed as the mean ± standard error (SE) of three different experiments. For each experiment, the number of samples in each group was 4 (*n* = 4 per each group). The fluorescence ratio or ATP level in untreated cells was set as 100%. * *p* < 0.05 vs. untreated cells; + *p* < 0.05 vs. cells with EtOH/POA stimulation alone (control).

To determine whether EtOH/POA induces mitochondrial dysfunction in AR42J cells, their MMPs and intracellular ATP levels were measured. MMP was measured by cytofluorimetric analysis using JC-1 fluorescent probe. The membrane potential-sensitive color shift is caused by the formation of red fluorescent J-aggregates. The green and red fluorescence images of the AR42J cells are shown in Figure 3A (left panel). The ratios of green to red fluorescence by the AR42J cells, reported in Figure 3A (right panel), reflect the relative cellular membrane potentials. EtOH/POA treatment decreased the MMP as demonstrated by the observed decrease in the ratio of red/green fluorescence (see Figure 3A right panel column “Untreated” vs column “Control”). Lycopene prevented the EtOH/POA-induced decrease in the ratio of red/green fluorescence, which indicates that lycopene blocks EtOH/POA-induced decrease in AR42J cell MMP (Figure 3A right panel; column “Lycopene” vs. column “Control”).

The data reported in Figure 3B show that the cellular ATP level decreased after EtOH/POA treatment (column “Untreated” vs column “Control”) and that pre-treatment with lycopene blocked this decrease (column “Lycopene” vs column “Control”) in a dose-dependent manner. Because lycopene arrested EtOH/POA-induced decreases in the MMP and ATP levels, it is evident that it can prevent EtOH/POA-induced mitochondrial dysfunction in AR42J cells.

### 2.4. Lycopene Inhibits EtOH/POA-Induced Zymogen Activation in AR42J Cells

**Figure 4:**
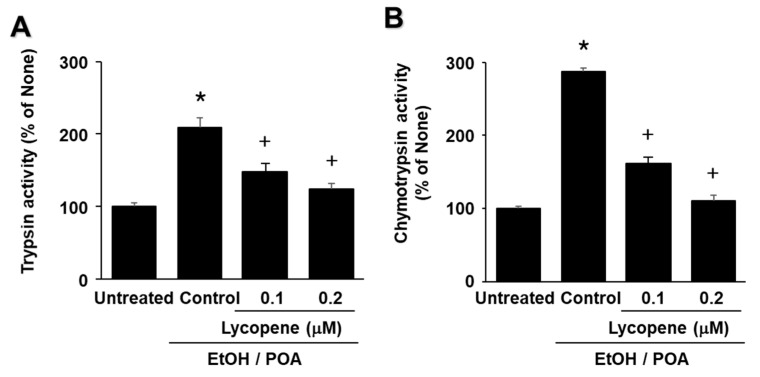
Lycopene inhibits ethanol and palmitoleic acid (EtOH/POA)-induced zymogen activation in AR42J cells. Cells were pretreated with the indicated concentrations of lycopene for 2 h and then stimulated with EtOH/POA for 20 min. (**A**) Trypsin activity was assayed using a fluorogenic assay with a trypsin-specific substrate. (**B**) Chymotrypsin activity was assayed using a fluorogenic assay with a chymotrypsin-specific substrate. The activity of trypsin or chymotrypsin in untreated cells was set as 100%. Data are expressed as the mean ± standard error (SE) of three different experiments. For each experiment, the number of samples in each group was 4 (*n* = 4 per each group). * *p* < 0.05 vs. untreated cells; + *p* < 0.05 vs. cells with EtOH/POA stimulation alone (control).

As shown in Figure 4A,B, EtOH/POA increased the activities of trypsin and chymotrypsin, which are markers of zymogen activation in pancreatic acinar cells. Lycopene decreased trypsin and chymotrypsin activities in EtOH/POA-stimulated cells in a dose-dependent manner. These results suggest that lycopene inhibits EtOH/POA-induced zymogen activation in AR42J cells.

### 2.5. Lycopene Inhibits EtOH/POA-Induced IL-6 Expression in AR42J Cells

**Figure 5:**
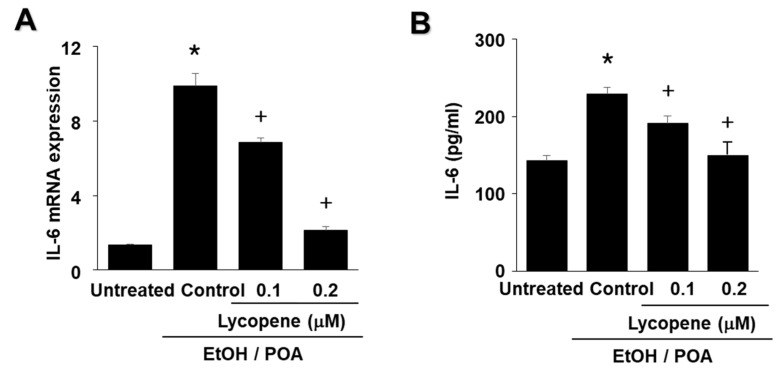
Lycopene inhibits ethanol and palmitoleic acid (EtOH/POA)-induced IL-6 expression in AR42J cells. Cells were pretreated with the indicated concentrations of lycopene for 2 h and then stimulated with EtOH/POA for 4 h (**A**) or 24 h (**B**). (**A**) mRNA expression of IL-6 was determined by real-time polymerase chain reaction (PCR) and normalized to β-actin. The IL-6 mRNA level in untreated cells was set as 1. (**B**) Level of IL-6 in the culture medium were measured by enzyme-linked immunosorbent assay (ELISA). Data are expressed as the mean ± standard error (SE) of three different experiments. For each experiment, the number of samples in each group was 4 (*n* = 4 per each group). * *p* < 0.05 vs. untreated cells; + *p* < 0.05 vs. cells with EtOH/POA stimulation alone (control).

Cells were treated with lycopene (0.1 μM or 0.2 μM) for 2 h, and then stimulated with EtOH/POA for 4 h (for mRNA expression, Figure 5A) or 24 h (for IL-6 levels in the medium, Figure 4B). EtOH/POA-induced mRNA expression of IL-6 was inhibited by lycopene, as determined by real-time reverse transcription polymerase chain reaction (RT-PCR) analysis (Figure 5A). Lycopene inhibited the EtOH/POA-induced increase in IL-6 levels in the medium in a dose-dependent manner (Figure 5B).

### 2.6. ML171 and NAC inhibit EtOH/POA-induced ROS production, NADPH oxidase activation, and NF-κB activation in AR42J cells

**Figure 6:**
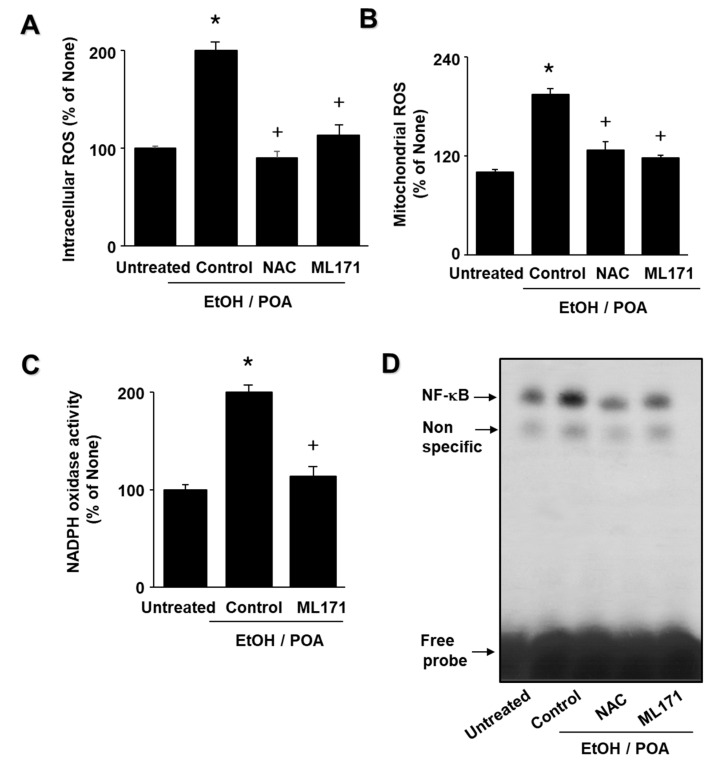
NADPH oxidase 1 inhibitor ML171 and antioxidant N-acetylcysteine (NAC) suppress ethanol and palmitoleic acid (EtOH/POA)-induced increases in intracellular and mitochondrial reactive oxygen species (ROS) and NADPH oxidase and NF-κB activation in AR42J cells. (**A**) The cells were pretreated with 2 μM ML171 or 1 mM NAC for 1 h, and then stimulated with EtOH/POA for 10 min (**A**–**C**) or 20 min (**D**). (**A**) Intracellular ROS levels were determined by dichlorofluorescein (DCF) fluorescence. (**B**) Mitochondrial ROS levels were measured by determining the level of fluorescent MitoSOX. (**C**) NADPH oxidase activity was determined by lucigenin assay. (**A**–**C**) ROS level or NADPH oxidase activity in untreated cells was set as 100%. Data are expressed as the mean ± standard error (SE) of three different experiments. For each experiment, the number of samples in each group was 4 (*n* = 4 per each group). * *p* < 0.05 vs. untreated cells; + *p* < 0.05 vs. cells with EtOH/POA stimulation alone (control). (**D**) DNA binding activities of NF-κB were determined using electrophoretic mobility shift assay (EMSA).

Because NADPH oxidase is known to play important roles in ROS production, we investigated the effects of the NADPH oxidase 1 (Nox1) inhibitor ML171, as well as the antioxidant NAC, on EtOH/POA-induced increases in intracellular and mitochondrial ROS levels. As shown in Figure 6A,B, ML171 and NAC decreased EtOH/POA-induced increases in intracellular and mitochondrial ROS levels. In addition, ML171 decreased EtOH/POA-induced increase in NADPH oxidase activity (Figure 6C). These results suggest that NADPH oxidase may mediate EtOH/POA-induced production of intracellular and mitochondrial ROS in AR42J cells. Furthermore, ML171 and NAC suppressed EtOH/POA-induced NF-κB activation (Figure 6D). Taken together, lycopene inhibits EtOH/POA-induced NF-κB activation by suppressing NADPH oxidase activation and ROS production in AR42J cells.

### 2.7. ML171 and NAC inhibit EtOH/POA-induced Zymogen Activation and IL-6 expression in AR42J cells

**Figure 7:**
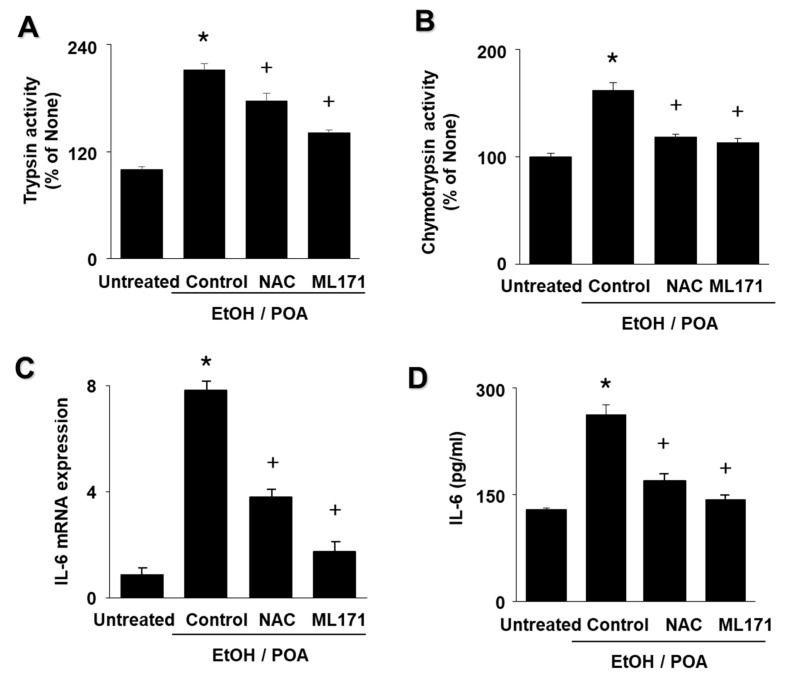
NADPH oxidase 1 inhibitor ML171 and antioxidant N-acetylcysteine (NAC) inhibit ethanol and palmitoleic acid (EtOH/POA)-induced zymogen activation and IL-6 expression in AR42J cells. The cells were pretreated with 2 μM ML171 or 1 mM NAC for 1 h, and then stimulated with EtOH/POA for 20 min (**A**,**B**), 4 h (**C**), and 24 h (**D**). (**A**) Trypsin activity was assayed using a fluorogenic assay with a trypsin-specific substrate. (**B**) Chymotrypsin activity was assayed using a fluorogenic assay with a chymotrypsin-specific substrate. (**C**) mRNA expression of IL-6 was determined by real-time polymerase chain reaction (PCR) and normalized to β-actin. (**D**) Levels of IL-6 in the culture medium were measured by enzyme-linked immunosorbent assay (ELISA). The activity of trypsin or chymotrypsin in untreated cells was set as 100% (**A**,**B**). IL-6 mRNA level in untreated cells was set as 1 (**C**). Data are expressed as the mean ± standard error (SE) of three different experiments. For each experiment, the number of samples in each group was 4 (*n* = 4 per each group). * *p* < 0.05 vs. untreated cells; + *p* < 0.05 vs. cells with EtOH/POA stimulation alone (control).

As shown in Figure 7A,B, EtOH/POA-induced activation of trypsin and chymotrypsin was inhibited by ML171 and NAC. ML171 and NAC decreased mRNA expression and protein levels of IL-6 in EtOH/POA-treated cells (Figure 7C,D). These results suggest that EtOH/POA-induced zymogen activation and IL-6 expression is induced by NADPH oxidase-mediated ROS production in AR42J cells.

## 3. Discussion

The present study was carried out to investigate whether lycopene can ameliorate inflammatory responses (IL-6 expression, zymogen activation) by reducing oxidative stress (intracellular and mitochondrial ROS, NADPH oxidase activity, NF-κB activation, mitochondrial dysfunction) in EtOH/POA-stimulated pancreatic acinar cells, which are an in vitro model of alcoholic pancreatitis. Several studies have identified that FAEEs, which are derived from nonoxidative alcohol metabolism, exert some detrimental effects in pancreatic acinar cells; these effects include increased pancreatic lysosomal fragility [24]. FAEEs also induce pancreatic zymogen activation, vacuolization of acinar cells, and significant increases in pancreatic edema, providing evidence that FAEEs can cause toxic pancreatic injury in vivo [25]. FAEE is formed in the presence of ethanol and free fatty acids. Treatment with EtOH/POA increased the levels of palmitoleic acid ethyl ester and induced acute pancreatic damage [7]. Thus, we used a cocktail of ethanol (final concentration of 150 mM) and palmitoleic acid (final concentration of 50 μM) to stimulate rat pancreatic acinar AR42J cells.

A high level of oxidative stress plays an important role in the initiation of alcoholic pancreatitis and has been considered as a therapeutic target for preventing acute pancreatitis. We previously showed that ROS mediate NF-κB activation and the expression of inflammatory cytokine IL-6 in cerulein-stimulated pancreatic acinar cells [26]. We also revealed that rebamipide, a small-molecule antioxidant, inhibited NF-κB activation and inflammatory cytokine production caused by phorbol myristate acetate-primed neutrophils in pancreatic acinar cells [27]. In this study, we found that NAC inhibited EtOH/POA-induced NF-κB activation, IL-6 expression, and zymogen activation in pancreatic acinar cells. These studies showed the relationship between ROS and NF-κB, which plays a critical role in the development of acute pancreatitis [28]. The present results suggest that ROS induce IL-6 expression and zymogen activation through NF-κB activation in EtOH/POA-stimulated AR42J cells. Since lycopene reduced intracellular and mitochondrial ROS in the present study, antioxidant activity of lycopene may contribute to inhibition of inflammatory responses including NF-κB activation, IL-6 expression, and zymogen activation as well as mitochondrial dysfunction in EtOH/POA-stimulated pancreatic acinar cells.

NADPH oxidase is known to be the source of ROS in pancreatic acinar cells during pancreatitis, and is considered to be potentially associated with pancreatitis [29,30]. To examine the effect of NADPH oxidase inhibition on ROS levels, we used the NADPH oxidase 1 inhibitor ML171. ML171 decreased ROS production in EtOH/POA-stimulated AR42J cells. In addition, EtOH/POA-induced NF-κB activation, zymogen activation, and IL-6 expression were suppressed by ML171 in AR42J cells. These results suggest a role for NADPH oxidase-mediated ROS production in NF-κB activation, zymogen activation, and IL-6 expression in EtOH/POA-stimulated AR42J cells.

Lycopene is one of the major carotenoids in Western diets and is found almost exclusively in tomatoes and tomato products [31]. It accounts for about 50% of carotenoids in human serum. Among the common dietary carotenoids, lycopene has the highest singlet oxygen quenching capacity in vitro [32]. Potential role of lycopene for human health is reported to be related to antioxidant activity of lycopene. In contrast to other carotenoids its serum values are not regularly reduced by smoking or alcohol consumption [33]. In this aspect, maintaining serum level of lycopene by dietary supplementation of lycopene may prevent alcoholic pancreatitis.

Regarding anti-inflammatory effect of lycopene in pancreas, lycopene inhibited oxidative stress and inflammatory cytokine expression, but increased GSH in pancreatic tissues in experimental pancreatitis models [22,23]. Patients with chronic pancreatitis had significantly lower plasma concentrations of antioxidants (selenium, vitamin A, vitamin E, beta-carotene, xanthine, beta-cryptoxanthine, and lycopene) compared with both control subjects and patients with recurrent acute pancreatitis [34]. Steenwijk et al. [35] screened the publications which were published before March 2020. They suggested that there is little evidence that increasing tomato intake or lycopene supplementation decreases inflammation. However, depletion of lycopene may be one of the first signs of low-grade inflammation. Therefore, it is beneficial to consume lycopene-rich foods occasionally to stay healthy and keep circulating lycopene at a basal level.

In the present study, we used AR42J cells which derive initially from a transplantable tumor of a rat exocrine pancreas [36]. This is the only cell line currently available that, in culture, maintains many characteristics of normal pancreatic acinar cells, such as the synthesis and secretion of digestive enzymes, protein expression, and proliferation [37,38]. AR42J cell receptor expression and signal transduction mechanisms parallel those of pancreatic acinar cells. Thus, this cell line has been widely used as an “in vitro” model to study the exocrine pancreas [39]. Further studies should be performed using primary pancreatic acinar cells in vitro as well as in vivo animal models to determine the inhibitory effect of lycopene on EtOH/POA-induced pancreatic inflammation and damage.

Regarding uptake of lycopene in cells, lycopene uptake is highly variable among cell lines [40,41]. In general, the initial uptake of lycopene is rapid, followed by a slower but sustained uptake. In LNCaP cells, an androgen-dependent prostate cancer cell line, lycopene (1. 48 μmol/L) was taken up from 1 h and saturated at 6 h [40]. Rapid uptake of lycopene by LNCaP cells might be facilitated by a receptor or binding protein and lycopene is stored selectively in the nucleus of LnCaP cells. In hepatic stellate cells, lycopene uptake increases until 24 h. It is distributed in the membrane fraction, cytosolic fraction, lipid droplets, and nuclei [41]. Based on these studies, we can postulate that lycopene may be taken up during 2 h-incubation in the present study. In humans, the primary storage site of lycopene is liver and high levels of lycopene have been detected in the adrenals, testes, and prostate [31]. Further study should be performed to determine the uptake efficiency and absorption mechanism of lycopene in pancreatic acinar cells.

For ROS determination, after 2 h-pre-treatment of lycopene, the cells were stimulated with EtOH/POA for 10 min. Based on the previous study [15], EtOH/POA treatment increased ROS levels in AR42J cells in a 10-min culture, which were slightly reduced in a 20-min culture [10]. Thus, the 10-min time point was used for the effect of lycopene on EtOH/POA-induced increases in ROS levels in the present study. Further study is necessary to monitor the ROS changes until 24 h in EtOH/POA-stimulated cells.

Even though lycopene inhibits NADPH oxidase activity in EtOH/POA-stimulated cells, ROS levels in both lycopene- and EtOH/POA-treated cells were still over the levels shown in untreated cells. We show that EtOH/POA treatment stimulates NADPH oxidase in AR42J cells. However, it may activate other ROS-producing cellular systems localized on the plasma membrane, in the cytosol, in the peroxisomes, in endoplasmic reticulum and so on. Further study is necessary to determine cellular sources of ROS in EtOH/POA-treated pancreatic acinar cells

We previously demonstrated that EtOH (100 mM)/POA (50 μM) did not affect cell viability until the 4-h culture, but induced cell death at 6 h in AR42J cells [15]. Low concentration of lycopene (<2 μM) had no effect on cell viability in SH-SY5Y neuroblastoma cells [42], colon cancer cells [43], and human hepatic adenocarcinoma SK-Hep-1 cells [44]. Therefore, lycopene may not induce cell death, but EtOH/POA may reduce cell viability after a 6-h culture in the present study. In the present study, we determined IL-6 protein levels at 24 h in EtOH/POA-stimulated cells. Further study should be performed to determine the relation of cell death and cytokine expression in EtOH/POA-treated pancreatic acinar cells.

In rats, lycopene treatment prevented decrease in GSH in pancreatic tissues in cerulein-induced pancreatitis [45] and L-arginine-induced pancreatitis [23]. Ali and Agha [46] showed that lycopene treatment prevented streptozotocin-induced decreases in activities of catalase (CAT), superoxide dismutase (SOD) and glutathione peroxidase (GPX) in pancreatic tissues in rats. They concluded that lycopene acts as an antidiabetic agent through lowering the free radicals and increases total antioxidant status with increased antioxidant enzyme activities (CAT, SOD, GPX). Taken together, lycopene reduces oxidative stress by maintaining antioxidant enzyme activities as normal levels. Its effect is mainly contributed by ROS scavenging activity of lycopene. Here, we found that lycopene inhibits EtOH/POA-induced activation and NADPH oxidase and thus, reduced ROS levels. Since EtOH/POA stimulation increases ROS levels, the antioxidant system (GSH, CAT, SOD, GPX) may be reduced for scavenging ROS in EtOH/POA-stimulated pancreatic acinar cells. Reduction of ROS by lycopene may prevent decreases in the antioxidant system (GSH, CAT, SOD, GPX) in EtOH/POA-stimulated pancreatic acinar cells. Further study is essential to determine whether lycopene maintains the levels of GSH, CAT, SOD, and GPX in EtOH/POA-stimulated pancreatic acinar cells.

In relation to in vitro and in vivo studies, Hammamoto et al. [6] determined FAEE synthase activity, FAEE content, and amylase activity were measured in pancreatic tissues of rats after 7 weeks of ethanol feeding. In ethanol-fed rats, FAEE content increased five-fold and amylase activity decreased up to 20% of that measured in the control group. FAEE content was inversely correlated with amylase activity. They suggested that FAEE may be responsible for the development of pancreatic damage by ethanol. Vigna et al. [47] induced alcoholic pancreatitis by injecting EtOH and POA in C57BL/6J mice. After 24 h, injection of EtOH and POA produced acute pancreatitis indicated by significant increases in histopathological damage and pancreatic myeloperoxidase concentrations. Therefore, lycopene supplementation may reduce EtOH/POA-induced pancreatic damage in vivo. Further study should be performed to determine the effect of lycopene on NADPH oxidase activity, ROS levels, mitochondrial function, NF-κB activity, IL-6 expression, and zymogen activation in pancreatic tissues using in vivo alcoholic pancreatitis models.

Lv et al. [22] determined anti-inflammatory effect of lycopene on severe acute pancreatitis (SAP) in both in vivo and in vitro models. They showed that lycopene reduced serum amylase, c-reactive protein, tumor necrosis factor-α, IL-6, macrophage inflammatory protein-1α, and monocyte chemotactic protein-1 in rats with SAP. In vitro pancreatic acinar cells, lycopene protected the cells against necrosis and apoptosis by relieving the mitochondrial and endoplasmic stress caused by ROS and decreasing active NF-κB p65. Previously, we showed that lycopene reduced IL-6 expression by inhibiting NF-κB activation in cerulein-stimulated pancreatic acinar cells [20]. In rats, lycopene treatment inhibited cerulein-induced oxidative stress (decreased GSH and increased myeloperoxidase activities) and reduced inflammatory cytokines tumor necrosis factor-α, and IL-1ß in pancreatic tissues [45]. In rats with L-arginine-induced pancreatitis, lycopene treatment reduced tumor necrosis factor-α, myeloperoxidase activity, and inducible nitric oxide synthase gene expression. Lycopene inhibited loss of pancreatic GSH in L-arginine treatment [23]. These studies suggest that antioxidant activity of lycopene contributes to anti-inflammatory effect in acute pancreatitis models. In the present study, lycopene reduced intracellular and mitochondrial ROS, mitochondrial dysfunction, and NF-kB activation and IL-6 expression in EtOH/POA-stimulated AR42J cells. Moreover, EtOH/POA increased NADPH oxidase activity in pancreatic acinar AR42J cells, which was inhibited by lycopene. Novelty of the present finding is that NADPH oxidase has a critical role to produce ROS in pancreatic acinar cells in the pathogenesis of alcoholic pancreatitis. Therefore, NADPH oxidase in pancreatic acinar cells may be therapeutic target for preventing or treating alcoholic pancreatitis. We show that lycopene has anti-inflammatory activity in EtOH/POA-induced IL-6 expression and zymogen activation by inhibiting NADPH oxidase in pancreatic acinar cells. Further in vivo studies are essential to support the present in vitro findings: the inhibitory effect of lycopene on pancreatic inflammation.

In the present study we show that lycopene inhibits NADPH oxidase activity and thus reduces ROS levels, leading to inhibition of NF-κB activation, IL-6 expression, and zymogen activation in EtOH/POA-stimulated cells. Since large amounts of ROS may induce mitochondrial dysfunction, as determined by decreased mitochondrial membrane potential and low ATP production, reducing ROS may be associated with preventing mitochondrial dysfunction in lycopene-treated pancreatic acinar cells exposed to EtOH/POA.

In conclusion, consumption of lycopene-rich foods may be beneficial for preventing or delaying the development of alcoholic acute pancreatitis by inhibiting NADPH oxidase-mediated ROS production and oxidative stress-induced inflammatory responses, including mitochondrial dysfunction, zymogen activation, and IL-6 expression in pancreatic acinar cells.

## 4. Materials and Methods

### 4.1. Cell Line and Culture Conditions

The rat pancreatic acinar cell line AR42J (pancreatoma; ATCC CRL 1492) was obtained from the American Type Culture Collection (Manassas, VA, USA). The cells were grown in Dulbecco’s modified Eagle’s medium (Sigma, St. Louis, MO, USA) supplemented with 10% fetal bovine serum (Gibco BRL, Grand Island, NY, USA) and antibiotics (100 U/mL penicillin and 100 μg/mL streptomycin). The cells were incubated at 37 °C in a humidified atmosphere of 95% air and 5% CO_2_.

### 4.2. Experimental protocol

Lycopene (L9879, Sigma-Aldrich) was dissolved in tetrahydrofuran (THF). AR42J cells (2.0 × 10^5^ /2 mL/well) were pre-treated with lycopene (final concentration, 0.1 or 0.2 μM) for 2 h and then treated with EtOH (150 mM)/POA (50 μM) for 10 min (to measure intracellular and mitochondrial ROS levels, NADPH oxidase activity, MMP, and ATP levels), 20 min (for analysis of NF-κB activation and zymogen activation), 4 h (to determine IL-6 mRNA expression), and 24 h (to measure IL-6 protein levels). To determine the involvement of NADPH oxidase and ROS, the cells were pretreated with the NADPH oxidase 1 inhibitor ML171 (2-Acetylphenothiazine, 2 μM) and NAC (1 mM) 1 h before EtOH/POA stimulation. AR42J cells incubated with THF (less than 0.5%) alone served as a control.

Our previous study showed that EtOH/POA treatment increased intracellular and mitochondrial ROS levels and NADPH oxidase activity, but decreased mitochondrial function at 10 min in AR42J cells [15]. Thus, intracellular and mitochondrial ROS levels, NADPH oxidase activity, and mitochondrial dysfunction were determined at 10 min after EtOH/POA treatment in the present study. To determine the appropriate incubation time for trypsin activity, NF-kB activation, and IL-6 expression, time-course experiments were performed after treatment of EtOH/POA in AR42J cells. AR42J cells (2.0 × 10^5^ /2 mL/well) were treated with EtOH (150 mM)/POA (50 μM) and cultured for 20, 40, and 60 min (for trypsin activity and NF-kB activity) or 1, 2, and 4 h (for IL-6 mRNA expression).

### 4.3. Preparation of Cell Extracts

The cells (1.0 × 10^6^ cells/10 mL/dish) in culture dishes were harvested by trypsin-ethylenediaminetetraacetic acid (EDTA) and pelleted by centrifugation at 1,000× *g* for 5 min. The pellets were resuspended in lysis buffer containing 25 mM Tris (pH 7.4), 150 mM NaCl, 1% Nonidet P-40 (NP-40), 0.5% sodium deoxycholate, 0.1% sodium dodecyl sulfate (SDS), and a commercial protease inhibitor complex (Complete; Roche, Mannheim, Germany). The cells were then lysed by drawing the cells through a 1-mL syringe with several rapid strokes. The mixture was then plated on ice for 30 min and centrifuged at 10,000× *g* for 15 min. The supernatants were collected and used as whole-cell extracts. To prepare membrane fractions, the supernatants were further centrifuged at 100,000× *g* for 1 h at 4 °C. The pellet was resuspended in lysis buffer containing 50 mM 4-(2-hydroxyethyl)-1-piperazineethanesulfonic acid (HEPES) (pH 7.4), 150 mM NaCl, 1 mM EDTA, and 10% glycerol to obtain the membrane fractions. For the preparation of nuclear extracts, the cells were lysed in hypotonic buffer containing 10 mM HEPES, 1.5 mM MgCl_2_, 10 mM KCl, 1 mM DL-dithiothreitol (DTT), 0.5 mM phenylmethylsulfonyl fluoride (PMSF), 0.05% NP-40, and 0.1 mM EDTA, followed by centrifugation at 10,000× *g* for 10 min. The nuclear pellets were resuspended in nuclear extraction buffer containing 20 mM HEPES (pH 7.9), 420 mM NaCl, 0.1 mM EDTA, 1.5 mM MgCl_2_, 25% glycerol, 1 mM DTT, and 0.5 mM PMSF and then centrifuged. The supernatants were collected and used as nuclear extracts. Protein concentration was determined by Bradford assay (Bio-Rad Laboratories, Hercules, CA, USA).

### 4.4. Measurement of Intracellular ROS Levels

The cells (2.0 × 10^5^ cells/2 mL/well) in 6-well plates were pre-treated with lycopene for 2 h and then treated with EtOH (150 mM)/POA (50 μM) for 10 min. The cells, which were treated with lycopene and EtOH/POA, were treated with 10 μg/mL dichlorofluorescein diacetate (DCF-DA; Sigma-Aldrich) and incubated in 5% CO_2_/95% air at 37 °C for 30 min. After incubation, the medium was removed and the cells were washed with PBS. The intensities of 2′,7′-dichlorofluorescein (DCF) fluorescence in the cells (in 6-well plates) were measured at 522 nm (excitation at 498 nm) with a Victor 5 multi-label counter (PerkinElmer Life and Analytical Sciences, Boston, MA, USA). Intracellular ROS levels were normalized to the cell numbers.

DCF-DA is used to detect intracellular ROS in live cells. DCF-DA is the most commonly used probe for measuring intracellular ROS. Cell-permeable DCF-DA diffuses into cells and is deacetylated by cellular esterases to form 2′,7′-dichlorodihydrofluorescein (H2DCF). In the presence of ROS, H2DCF is rapidly oxidized to 2′,7′-dichlorofluorescein (DCF), which is highly fluorescent, with excitation and emission wavelengths of 498 and 522 nm, respectively. The DCF-DA assay can provide reliable measurements of ROS levels in cells [48].

### 4.5. Measurement of mitochondrial ROS levels

The cells (2.0 × 10^5^ cells/2 mL/well) in 6-well plates were treated with lycopene for 10 min. To assess mitochondrial ROS levels, the cells were treated with 10 µM MitoSOX (Life Technologies, Grand Island, NY, USA) for 30 min, before being washed and scraped into phosphate-buffered saline (PBS). The intensity of MitoSOX fluorescence at 585 nm (excitation at 524 nm) was measured with a Victor 5 multi-label counter (PerkinElmer Life and Analytical Sciences). The mitochondrial ROS levels were normalized to the cell numbers, and ROS levels were assessed based on equal cell numbers.

### 4.6. Measurement of MMP

To determine changes in MMP, the cells (1.0 × 10^6^ cells/10 mL/dish) in culture dishes were cultured on glass coverslips coated with poly-L-lysine and pretreated with lycopene for 10 min. The cells were then incubated with 5,5,6,6-tetrachloro-1,1,3,3-tetraethyl benzimidazolyl carbocyanine iodide (JC-1) reagent (1:100; 10009908, Cayman Chemical Company, Ann Arbor, MI, USA) for 20 min. After removing the media, the cells were dried for 15 min at 20–22 °C, washed twice with PBS for 5 min, and mounted with mounting solution (M-7534, Sigma Aldrich, St. Louis, MO, USA). Fluorescence of JC-1 in the cells was determined (red; excitation at 590 nm and emission at 610 nm, green; excitation at 485 nm and emission at 535 nm) using a laser-scanning confocal microscope (LSM 880, Carl Zeiss Inc., Oberkochen, Germany). The fluorescence images were expressed as the percentage ratio of red and green intensities using NIH ImageJ 5.0 software (National Institutes of Health, Bethesda, MD, USA).

### 4.7. Measurement of ATP Levels

ATP levels were measured using a luminescent ATP detection assay kit according to the manufacturer’s protocol (ab113849; Abcam, Cambridge, UK). The cells (2.0 × 10^5^ cells/2 mL/well) were stimulated in 6-well plates with EtOH/POA for 10 min, and then substrate buffer was added to the lyophilized ATP substrate to prepare the luminescent substrate solution. Luminescence was measured using a microplate reader (Molecular Devices, Sunnyvale, CA, USA). The relative ATP concentration was determined by interpolation within the ATP standard reference.

### 4.8. Measurement of NADPH Oxidase Activity

NADPH oxidase activity was measured by lucigenin assay. To measure the NADPH oxidase activity in the membrane extracts, the cells were stimulated with EtOH/POA for 10 min. The assay was performed in 50 mM Tris-N-morpholino)ethanesulfonic acid (MES) buffer (pH 7.0) containing 2 mM potassium cyanide (KCN), 10 μM Lucigenin, and 100 μM NADPH. The reaction was initiated by adding 10 μg of membrane protein extract. Photon emission was measured using a Victor5 multi-label counter (PerkinElmer Life and Analytical Sciences). Cytosolic protein extract was used in place of membrane protein extract as a control.

### 4.9. Electrophoretic Mobility Shift Assay (EMSA)

The cells were treated with lycopene for 20 min. Nuclear extracts (2 µg) of the cells were incubated with the ^32^P-labeled double-stranded oligonucleotide 5′-GGGCCAAGAATCTTAGCAGTTTCGGG-3′ in buffer containing 12% glycerol, 12 mM HEPES (pH 7.9), 1 mM EDTA, 1 mM DTT, 25 mM KCl, 5 mM MgCl_2_, and 0.04 µg/mL poly[d(I-C)] at room temperature for 30 min. The extracts were then subjected to electrophoretic separation at room temperature on a non-denaturing 5% acrylamide gel at 30 mA using 0.5× Tris borate/EDTA buffer. The gels were dried at 80 °C for 1 h and exposed to radiography film for 24 h at −70 °C with intensifying screens.

### 4.10. Assays for Enzyme Activities

The cells (1.0 × 10^6^ cells/10 mL/dish) in culture dishes were treated with EtOH/POA for 20 min, harvested by trypsin–EDTA, and pelleted by centrifugation at 1000× *g* for 5 min. The pellets were washed with PBS and then resuspended in assay buffer containing 50 mM Tris (pH 8.0), 150 mM NaCl, 1 mM CaCl_2_, and 0.1% bovine serum albumin (BSA) and lysed by drawing the cells through a 1-mL syringe with several rapid strokes. The mixture was then centrifuged at 10,000× *g* for 2 min. Supernatants were used for the assay. Trypsin and chymotrypsin activity were measured fluorometrically (excitation at 380 nm and emission at 440 nm) with a Victor5 multilabel counter. Trypsin activity was measured using a fluorogenic assay with a substrate specific for trypsin (Boc-Glu-Ala-Arg-AMC) (MQR-3135-v, Peptides International, Louisville, KY, USA). Chymotrypsin activity was measured using a fluorogenic assay with a substrate specific for chymotrypsin (Suc-Ala-Ala-Pro-Phe-MCA) (MAA-3114-v, Peptides International).

### 4.11. Real-Time PCR Analysis for IL-6

The cells (2.0 × 10^5^ cells/2 mL/well) were treated with lycopene in 6-well plates for 4 h. Total RNA was isolated using TRI reagent ® (Molecular Research Center, Inc., Cincinnati, OH, USA). Total RNA was converted into cDNA by reverse transcription with a random hexamer and MuLV reverse transcriptase (Promega, Madison, WI, USA) and heating at 23 °C for 10 min, 37 °C for 60 min, and 95 °C for 5 min. The cDNA was used for real-time PCR with specific primers for IL-6 and β-actin. The sequences of the IL-6 primers used to produce the desired 590 bp PCR products were 5′- GAGAGGAGACTTCACAGAGGATACCA-3′ (forward primer) and 5′-CCACAGTGAGGAATGTCCACAA-3′ (reverse primer). For β-actin cDNA production, the 349-bp PCR product was obtained using the forward primer 5′-ACCAACTGGGACGACATGGAG-3′ and reverse primer 5′-GTGAGGATCTTCATGAGGTAGTC-3′. For PCR amplification, the cDNA was amplified by 35 repeat denaturation cycles at 95 °C for 30 s, annealing at 53 °C for 30 s, and extension at 72 °C for 45 s. During the first cycle, the 95 °C step was extended to 3 min. The β-actin gene was amplified in the same reaction to serve as the reference gene.

### 4.12. Enzyme-Linked Immunosorbent Assay (ELISA) for IL-6

The cells (2.0 × 10^5^ cells/2 mL/well) in 6-well plates) were treated with lycopene for 24 h. The concentration of IL-6 in the medium was determined using enzyme-linked immunosorbent assay kits (R&D Systems, Minneapolis, MN, USA) according to the manufacturer’s instructions.

### 4.13. Statistical Analysis

One-way analysis of variance (ANOVA) followed by Newman–Keul’s post hoc test, was used for statistical analysis. All data are reported as the mean ± standard error (SE) of three different experiments. For each experiment, the number of samples in each group was 4 (*n* = 4 per each group). A *p*-value of 0.05 was considered statistically significant.

## Data Availability

The data used to support the findings of this study are included within the article.
